# The most widely viewed YouTube videos with content related to multivitamins

**DOI:** 10.15171/hpp.2016.35

**Published:** 2016-10-01

**Authors:** Corey H. Basch, Jennifer Mongiovi, Alyssa Berdnik, Charles E. Basch

**Affiliations:** ^1^Department of Public Health, William Paterson University, New Jersey, USA; ^2^Mailman School of Public Health, Columbia University, New York, NY, USA; ^3^Department of Health and Behavior Studies, Teachers College, Columbia University, New York, NY, USA

**Keywords:** YouTube, Multivitamins, Social media

## Abstract

**Background:** Use of multivitamin/multimineral (MVM) and other dietary supplements is common among American adults. The purpose of this study was to describe the source and content of the most widely viewed YouTube videos associated with MVM supplements.

**Methods:** Videos were filtered by number of views and the source of the video upload was recorded. A comparison of video characteristics and differences in video content was conducted.

**Results:** Cumulatively, the videos in this sample were viewed 25 573 055 times. The majority of videos found in this sample were uploaded by a nutrition, wellness, or fitness channels. Most videos mentioned benefits (80.4%, 95% CI: 72.5%, 88.3%) and advocated for use of the supplement (72.2%, 95% CI: 63.3%, 81.1%). Over 84% (84.5%, 95% CI: 77.3, 91.7%) of the videos did not mention risks associated with taking a particular vitamin or supplement.

**Conclusion:** The findings of this study indicate that MVMs are often promoted and encouraged, yet risks associated with MVMs were infrequently mentioned. Health professionals should be aware of the extent to which MVM related content appears on social media and, more importantly, be attuned to the content, which can be misleading, or missing information regarding risks and/or evidence of possible benefits.

## Introduction

Over 50% of American adults have reported use of dietary supplements, the majority of which are multivitamin/multimineral (MVM) supplements.^1,2^ Supplements, vitamins, and preventive care are of particular interest among the Millennial generation, currently between 18 and 36 years of age, who have been driving sales in this market.^3^ These young to middle-aged adults, especially women, are often at risk for inadequate micronutrient intake due to demanding lifestyles, poor dietary behaviors, and attempts to lose weight.^4^ The health benefits of taking supplements are not universal and vary both by type and person.^5^

Among this generation, healthy behaviors are often encouraged through the use of various wearable devices, mobile apps, and social media programs.^6^ Social media and sharing sites encourage are increasingly being sought as sources of health information.^7^ Between 50%-60% of individuals living in North America rely on the Internet for health information, however, minimal research has been done regarding health information presented on social media and sharing sites.^7,8^

YouTube.com™ is a popular social media platform with over a billion users.^9^ To our knowledge, there are no published papers regarding the content of videos published on this platform related to MVM supplements. Hence, the purpose of this study was to describe the source and content of the most widely viewed YouTube videos associated with MVM supplements. 

## Materials and Methods

A search of YouTube.com™ was conducted using the following search terms, “multivitamin supplement” and “vitamin.” Of the 100 most viewed videos (determined by filtering and sorting by the number of views on YouTube, only those that were spoken in English and featured products for human use were used in analysis. One video was excluded because it was not in English and two were excluded, as their product was intended for an animal. Each video was evaluated and coded for number of views, length in minutes, upload source, and content.

Upload sources were defined as follows: (1) Nutrition, wellness, or fitness channel – video uploaded by a YouTube.com™ channel with content focused on nutrition, wellness, or fitness (including body building); (2) Television/Internet—A clip that was uploaded by the network; or Internet-based news or programming; (3) Consumer—a clip uploaded by an individual without any professional affiliation; (4) Company channel or product advertisement – any video uploaded by a supplement company or promoting product sales; (5) Medical professional – video uploaded by a doctor of medicine (MD) or registered nurse (RN). One researcher (AB) sorted all videos and analyzed the entire sample. A random number generator was used to select 10% of videos to be coded by a second researcher (CHB). A kappa of 0.98 indicated a very high level of agreement between reviewers. If human subjects are not included in the research, review by the institutional review boards at William Paterson University and at Teachers College is not required.

Descriptive statistics included calculating frequencies, percentages, means, and ranges, additionally 95% CI of the measures were provided. Analysis of variance (ANOVA) was used to compare video characteristics and differences in video content were examined using chi-square analysis. Analyses were conducted using IBM SPSS (version 22, IBM Corp., Armonk, NY USA). *P*<0.05 was considered statistically significant.

## Results

Together, the 97 videos analyzed were viewed 25 573 055 times. The majority of videos found in this sample were uploaded by a nutrition, wellness, or fitness channels. The mean length of all videos was nearly 11 minutes and, on average, viewed over 200 000 times (Table 1). No significant differences were found in the mean number of views between upload sources. The mean length in minutes was highest among videos uploaded by television/internet sources (20.0 [26.9]) and shortest among videos uploaded by vitamin company channels or advertisements (4.0 [3.2], *P* = 0.01). Most videos mentioned benefits (80.4%, 95% CI: 72.5%, 88.3%) and advocated for use of the supplement (72.2%, 95% CI: 63.3%, 81.1%) (Table 1). Over 84% (84.5%, 95% CI: 77.3, 91.7%) of the videos did not mention risks associated with taking a particular vitamin or supplement, but 42.9% (95% CI: 24.6%, 61.2%) of videos uploaded by television and internet sources mentioned such risks. More than half of the videos uploaded by television and internet sources (60.7%, 95% CI: 42.6%, 78.8%) and by medical professionals (55.6%, 95% CI: 23.1%, 88.1%) referred to previous studies or research, while less than half of the videos uploaded from other sources referred to research, with only one in four company channel videos and advertisements referring to existing research.

**Table 1:** Characteristics of 97 YouTube.com‏™ videos on multivitamin supplements and vitamins

	**Upload source**						***P*** ** value**
	**Total (n=97)**	**Nutrition, wellness, or fitness channel (n=31)**	**Television/Internet (n=28)**	**Consumer (n=17)**	**Vitamin company channel/advertisement (n=12)**	**Medical professional (n=9)**	
Mean number of views (SD)	263639.7 (940641.9)	306408.4 (697921.6)	405301.2 (1591739.4)	131345.1 (107589.8)	83888.6 (50242.4)	165159.2 (198868.7)	0.82
95% CI number of views	76448.1, 450831.4	60726.0, 552090.7	349097.2, 461505.2	80201.1, 182489.1	55461.8, 112315.4	35234.0, 295084.4	
Range number of views	39312- 8507748	43775- 3933582	44431- 8507748	39327- 341330	42077-201528	39312-592300	
Mean length in min (SD)	10.9 (16.9)	6.6 (3.0)	20.0 (26.9)	7.8 (11.7)	4.0 (3.2)	12.6 (14.5)	0.10
95% CI length in min	7.5, 14.3	5.6, 7.7	10.0, 29.9	2.2, 13.3	2.2, 5.8	3.1, 22.1	
Range length in min	0.6-88.8	2.4-15.2	2.3-88.8	0.6-51.8	0.7-9.2	4.0-50.6	
**Content**	**n (%)**	**n (%)**	**n (%)**	**n (%)**	**n (%)**	**n (%)**	
Refers to studies/research	44 (45.4)	14 (45.2)	17 (60.7)	5 (29.4)	3 (25.0)	5 (55.6)	0.15
Mentions how much (units) to take	41 (42.3)	15 (48.2)	15 (53.6)	5 (29.4)	3 (25.0)	3 (33.3)	0.3
Mentions benefits	78 (80.4)	25 (80.6)	24 (85.7)	14 (82.4)	8 (66.7)	6 (66.7)	0.73
Advocates use of the mentioned supplement(s)	70 (72.2)	23 (74.2)	18 (64.3)	14 (82.4)	9 (75.0)	6 (66.7)	0.73
Mentions that it is safe	8 (8.2)	4 (12.9)	2 (7.1)	2 (11.8)	0.0)	0 (0.0)	0.54
Mentions risk of taking supplement	15 (15.5)	1 (3.2)	12 (42.9)	0 (0.0)	0 (0.0)	2 (22.2)	<0.0001

## Conclusions

This study is novel in that it describes the content of the most popular videos on YouTube.com™ focused on MVM supplements. The findings of this study indicate that MVMs are often promoted and encouraged, yet risks associated with MVMs were infrequently mentioned. Advertising for vitamins and supplements has been prominent since the 1930s even though their promotions are often offering false hopes.^10^ Popular news media and advertisements promote vitamins as crucial for two reasons (1) to help ensure sufficient dietary intake of micro-nutrients and (2) to help enhance beauty.^10^ Advertising also promotes the idea that vitamin supplements are required for optimal health.

While today’s vitamin industry represents income in the billions, questions arise about how the industry and supplements are being regulated.^10^ The Federal Trade Commission (FTC) enforces laws that ban “unfair or deceptive acts or practices” in the United States.^11^ and the FTC plays a role in ensuring that “customers get accurate information about dietary supplements so that they can make informed decisions about these products.”^11^ The FTC also has primary responsibility for claims in advertising and direct marketing materials, for example that advertising for vitamins and dietary supplements are truthful and not misleading. This type of regulation does not apply to content from independent sources, such as content uploaded by consumers on public sharing platforms that reflect opinions that may be mistaken for fact.

In addition to the FTC, other organizations attempt to promote truthful marketing in the vitamin supplement industry. The Council for Responsible Nutrition Foundation (CRNF) has pledged grants to the National Advertising Division (NAD), a service of the Council of Better Business Bureaus (CBBB), to monitor advertising for dietary supplements in a self-regulatory program.^11^ The program conducted by NAD allows companies to voluntarily alter actions that are not compliant behavior prior to facing potential consequences from law enforcement agencies like the FTC.^12^

The limitations of this study include the cross-sectional design (popularity based on number of views changes constantly), and the inclusion of only the 100 most widely viewed videos (an arbitrary cut point). Nevertheless, this study contributes to the literature about an emerging topic, namely how social media may influence consumers’ decision-making related MVM use. Health professionals should be aware of the extent to which MVM related content appears on social media and, more importantly, be attuned to the content, which can be misleading, or missing information regarding risks and/or evidence of possible benefits.

## Ethical approval

This study was exempt from requiring IRB approval since no data from human subjects was used.

## Competing interests

The authors declare that they have no competing interests.

## Funding

No funding was received for this study.

## Authors’ contributions

CHB and CEB conceptualized the study, AB collected the data, JM analyzed the data. All authors contributed to writing this manuscript. 

## References

[1] Velicer CM, Ulrich CM (2008). Vitamin and mineral supplement use among US adults after cancer diagnosis: a systematic review. J Clin Oncol.

[2] National Institutes of Health State-of-the-Science Panel (2007). National Institutes of Health State-of-the-Science Conference Statement: multivitamin/mineral supplements and chronic disease prevention. Am J Clin Nutr.

[3] Vitamins, Minerals & Supplements: The Role of the Physician. AccentHealth; 2014. http://www.accenthealth.com/AccentHealth/media/Documents/VMS_whitepaper_2014.pdf.

[4] Huskisson E, Maggini S, Ruf M (2007). The role of vitamins and minerals in energy metabolism and well-being. J Int Med Res.

[5] Harvie M. Nutritional supplements and cancer: potential benefits and proven harms. Am Soc Clin Oncol Educ Book. 2014:e478-86. doi:10.14694/EdBook_AM.2014.34.e478. 24857143

[6] D’Adamo A. Opportunities in the Vitamin and Supplement Industry. Women’s Marketing; 2015. Available from: http://www.womensmarketing.com/blog/2015/06/opportunities-in-the-vitamin-and-supplement-industry/.

[7] Knight E, Intzandt B, MacDougall A, Saunders TJ (2015). Information seeking in social media: a review of YouTube for sedentary behavior content. Interact J Med Res.

[8] Thackeray R, Crookston BT, West JH (2013). Correlates of health-related social media use among adults. J Med Internet Res.

[9] YouTube (n.d.). Statistics. https://www.youtube.com/yt/press/statistics.html.

[10] Ward JW, Warren C, editors. Silent victories: The History and Practice of Public Health in Twentieth Century America. New York, NY: Oxford University Press; 2006. p. 194-204.

[11] Federal Trade Comission. Dietary Supplements: An Advertising Guide for Industry. Available from: https://www.ftc.gov/system/files/documents/plain-language/bus09-dietary-supplements-advertising-guide-industry.pdf. 10.1080/20014091091742 11221600

[12] The Council for Responsible Nutrition. CRN foundation renews its commitment to self-regulatory efforts for dietary supplement advertising: CRNF extends funding for NAD program through 2017. 2014. Available form: http://www.crnusa.org/CRNfoundation/CRNFPR15-NAD011215.pdf.

